# Supplementary material for: Trajectories in muscular strength and physical function among men with and without prostate cancer in the health aging and body composition study
Source: PLoS One. 2020 Feb 13;15(2):e0228773. doi: 10.1371/journal.pone.0228773 (PMC7017990; doi:10.1371/journal.pone.0228773)
Supplement: S1 Table — (DOCX) [file pone.0228773.s001.docx]

### S1 Table. Characteristics of the study sample at last pre-diagnosis (index) visit

|  | **PC Cases (N=117)**  **Mean (SD) or n (%)** | **Controls (n=468)**  **Mean (SD) or n (%)** | **p-value** |
| --- | --- | --- | --- |
| **Time from index visit to diagnosis (months)** | 12.0 (8.4) | 0 |  |
| **Age at index visit** | 76.4 (3.6) | 76.6 (3.5) | 0.521 |
| **Index visit** |  |  | 0.998 |
| Baseline (year 1) | 25 (21.4%) | 96 (20.5%) |  |
| Year 2 | 39 (33.3%) | 156 (33.3%) |  |
| Year 4 | 18 (15.4%) | 72 (15.4%) |  |
| Year 6 | 26 (22.2%) | 104 (22.2%) |  |
| Year 8 | 9 (7.7%) | 40 (8.5%) |  |
| **Race** |  |  | 0.967 |
| White | 58 (49.6%) | 231 (49.4%) |  |
| Black | 59 (50.4%) | 237 (50.6%) |  |
| **Education** |  |  | 0.076 |
| Less than HS | 32 (27.4%) | 157 (33.5%) |  |
| HS grad | 26 (22.2%) | 129 (27.6%) |  |
| Postsecondary | 59 (50.4%) | 182 (38.9%) |  |
| **Married** |  |  | 0.121 |
| No | 42 (35.9%) | 131 (28.5%) |  |
| Yes | 75 (64.1%) | 328 (71.5%) |  |
| **Diabetes** |  |  | 0.730 |
| No | 89 (76.1%) | 363 (77.6%) |  |
| Yes | 28 (23.9%) | 105 (22.4%) |  |
| **Heart attack** |  |  | 0.023 |
| No | 101 (86.3%) | 358 (76.7%) |  |
| Yes | 16 (13.7%) | 109 (23.3%) |  |
| **Hypertension/high BP** |  |  | 0.894 |
| No | 52 (44.8%) | 213 (45.5%) |  |
| Yes | 64 (55.2%) | 255 (54.5%) |  |
| **Stroke** |  |  | 0.162 |
| No | 113 (97.4%) | 439 (94.2%) |  |
| Yes | 3 (2.6%) | 27 (5.8%) |  |
| **CHF** |  |  | 0.398 |
| No | 111 (96.5%) | 438 (94.6%) |  |
| Yes | 4 (3.5%) | 25 (5.4%) |  |
| **Number of comorbidities** ^A^ | 1.0 (0.8) | 1.1 (0.9) | 0.173 |
| **Arthritis** |  |  | 0.296 |
| No | 55 (47.0%) | 195 (41.7%) |  |
| Yes | 62 (53.0%) | 273 (58.3%) |  |
| **Cancer** ^B^ |  |  | **<.001** |
| No | 99 (84.6%) | 468 (100.0%) |  |
| Yes | 18 (15.4%) | 0 (0.0%) |  |
| **BMI** | 26.7 (3.7) | 27.0 (4.1) | 0.463 |
| **% Body Fat** | 29.2 (4.5) | 29.3 (5.4) | 0.971 |
| **Lean Body Mass (Kg)** | 54.0 (67.4) | 54.0 (69.9) | 0.929 |
| **CESD** | 5.6 (5.0) | 5.9 (6.1) | 0.701 |
| **Falls in last 12 months** |  |  | 0.334 |
| No | 86 (74.1%) | 365 (78.3%) |  |
| Yes | 30 (25.9%) | 101 (21.7%) |  |
| **Easy walking a quarter mile** |  |  | 0.107 |
| No (unable to do – not that easy) | 16 (14.0%) | 95 (20.7%) |  |
| Yes (easy or somewhat easy) | 98 (86.0%) | 364 (79.3%) |  |
| **Easy walking up 10 steps** |  |  | 0.850 |
| No (unable to do – not that easy) | 17 (15.2%) | 66 (14.5%) |  |
| Yes (easy or somewhat easy) | 95 (84.8%) | 390 (85.5%) |  |
| **Easy lifting/carrying 10 pounds** |  |  | 0.188 |
| No (unable to do – not that easy) | 4 (3.7%) | 31 (7.1%) |  |
| Yes (easy or somewhat easy) | 105 (96.3%) | 404 (92.9%) |  |
| **Past 12 months any high intensity exercise** |  |  | 0.338 |
| No | 74 (67.9%) | 314 (72.5%) |  |
| Yes | 35 (32.1%) | 119 (27.5%) |  |
| **Past 7 days any high intensity exercise** |  |  | 0.683 |
| No | 89 (81.7%) | 346 (79.9%) |  |
| Yes | 20 (18.3%) | 87 (20.1%) |  |
| **Did 400m walk** |  |  | 0.197 |
| No | 31 (26.5%) | 153 (32.7%) |  |
| Yes | 86 (73.5%) | 315 (67.3%) |  |
| **20m gait speed (m/sec** | 1.3 (0.2) | 1.3 (0.4) | 0.517 |
| **HABCPPB** | 2.2 (0.6) | 2.1 (0.6) | 0.253 |
| **Grip strength** ^C^ | 42.2 (9.8) | 39.4 (8.5) | 0.004 |
| **Isokinetic quad strength** | 311.3 (109.2) | 296.2 (104.5) | 0.209 |

Note: PC, Prostate Cancer; CHF, Chronic Heart Failure; BMI, Body Mass Index; CESD, Center for Epidemiologic Studies Depression Scale; HABCPPB, Health Aging and Body Composition Physical Performance Battery; ^A^ diabetes, heart attack, hypertension/high blood pressure, stroke, CHF; ^B^ by design of our sample there are no cancer in control group, ^C^ max of two hands
