# Supplementary material for: Trajectories in muscular strength and physical function among men with and without prostate cancer in the health aging and body composition study
Source: PLoS One. 2020 Feb 13;15(2):e0228773. doi: 10.1371/journal.pone.0228773 (PMC7017990; doi:10.1371/journal.pone.0228773)
Supplement: S2 Table — (DOCX) [file pone.0228773.s002.docx]

### S2 Table. Change in grip strength at 3-4 year follow-up visit

|  | **Decreasing**  **Mean (SD) or n (%)** | **Consistently Low**  **Mean (SD) or n (%)** | **Consistently High**  **Mean (SD) or n (%)** | **Increasing**  **Mean (SD) or n (%)** | **p-value** |
| --- | --- | --- | --- | --- | --- |
| **Age at index visit** | 76.0 (3.2) | 77.1 (3.6) | 74.5 (2.7) | 76.7 (3.9) | 0.034 |
| **Years since index visit** |  |  |  |  | 0.151 |
| **3** | 4 (22.2%) | 4 (13.8%) | 10 (38.5%) | 0 (0.0%) |  |
| **4** | 14 (77.8%) | 25 (86.2%) | 16 (61.5%) | 2 (100.0%) |  |
| **Race** |  |  |  |  | 0.317 |
| White | 11 (61.1%) | 14 (48.3%) | 11 (42.3%) | 2 (100.0%) |  |
| Black | 7 (38.9%) | 15 (51.7%) | 15 (57.7%) | 0 (0.0%) |  |
| **Education** |  |  |  |  | 0.267 |
| Less than HS | 2 (11.1%) | 8 (27.6%) | 3 (11.5%) | 0 (0.0%) |  |
| HS grad | 4 (22.2%) | 10 (34.5%) | 6 (23.1%) | 0 (0.0%) |  |
| Postsecondary | 12 (66.7%) | 11 (37.9%) | 17 (65.4%) | 2 (100.0%) |  |
| **Married** |  |  |  |  | 0.212 |
| No | 7 (38.9%) | 11 (37.9%) | 4 (15.4%) | 1 (50.0%) |  |
| Yes | 11 (61.1%) | 18 (62.1%) | 22 (84.6%) | 1 (50.0%) |  |
| **Diabetes** |  |  |  |  | 0.685 |
| No | 14 (77.8%) | 23 (79.3%) | 18 (69.2%) | 2 (100.0%) |  |
| Yes | 4 (22.2%) | 6 (20.7%) | 8 (30.8%) | 0 (0.0%) |  |
| **Heart Attack** |  |  |  |  | 0.942 |
| No | 16 (88.9%) | 26 (89.7%) | 24 (92.3%) | 2 (100.0%) |  |
| Yes | 2 (11.1%) | 3 (10.3%) | 2 (7.7%) | 0 (0.0%) |  |
| **Hypertension/High BP** |  |  |  |  | 0.564 |
| No | 11 (61.1%) | 12 (41.4%) | 11 (42.3%) | 1 (50.0%) |  |
| Yes | 7 (38.9%) | 17 (58.6%) | 15 (57.7%) | 1 (50.0%) |  |
| **Stroke** |  |  |  |  | 0.337 |
| No | 18 (100.0%) | 26 (92.9%) | 26 (100.0%) | 2 (100.0%) |  |
| Yes | 0 (0.0%) | 2 (7.1%) | 0 (0.0%) | 0 (0.0%) |  |
| **CHF** |  |  |  |  | 0.666 |
| No | 17 (100.0%) | 28 (96.6%) | 26 (100.0%) | 2 (100.0%) |  |
| Yes | 0 (0.0%) | 1 (3.4%) | 0 (0.0%) | 0 (0.0%) |  |
| **Number of Comorbidities** ^A^ | 0.7 (0.7) | 1.0 (0.9) | 1.0 (0.7) | 0.5 (0.7) | 0.555 |
| **Arthritis** |  |  |  |  | 0.484 |
| No | 9 (50.0%) | 13 (44.8%) | 14 (53.8%) | 2 (100.0%) |  |
| Yes | 9 (50.0%) | 16 (55.2%) | 12 (46.2%) | 0 (0.0%) |  |
| **Cancer** ^B^ |  |  |  |  | 0.506 |
| No | 15 (83.3%) | 23 (79.3%) | 23 (88.5%) | 1 (50.0%) |  |
| Yes | 3 (16.7%) | 6 (20.7%) | 3 (11.5%) | 1 (50.0%) |  |
| **BMI** | 26.8 (4.3) | 25.3 (2.8) | 27.2 (3.3) | 25.6 (1.1) | 0.223 |
| **% Body Fat** | 29.5 (3.8) | 28.3 (3.8) | 28.9 (4.1) | 30.1 (1.2) | 0.761 |
| **Lean Body Mass (Kg)** | 54.5 (79.8) | 51.4 (70.8) | 56.1 (53.0) | 46.1 (30.5) | **0.029** |
| **CESD** | 3.5 (2.4) | 8.1 (6.3) | 3.4 (4.0) | 12.0 (.) | **0.010** |
| **Falls in last 12 months** |  |  |  |  | 0.432 |
| No | 12 (66.7%) | 21 (72.4%) | 22 (84.6%) | 2 (100.0%) |  |
| Yes | 6 (33.3%) | 8 (27.6%) | 4 (15.4%) | 0 (0.0%) |  |
| **Easy walking a quarter mile** |  |  |  |  | 0.747 |
| No | 1 (5.6%) | 4 (13.8%) | 2 (8.0%) | 0 (0.0%) |  |
| Yes | 17 (94.4%) | 25 (86.2%) | 23 (92.0%) | 2 (100.0%) |  |
| **Easy lifting/carrying 10 pounds** |  |  |  |  | 0.388 |
| No | 1 (5.6%) | 3 (10.3%) | 0 (0.0%) | 0 (0.0%) |  |
| Yes | 17 (94.4%) | 26 (89.7%) | 26 (100.0%) | 2 (100.0%) |  |
| **Past 12 months... high intensity exercise** |  |  |  |  | 0.408 |
| No | 11 (61.1%) | 23 (79.3%) | 16 (61.5%) | 1 (50.0%) |  |
| Yes | 7 (38.9%) | 6 (20.7%) | 10 (38.5%) | 1 (50.0%) |  |
| **Past 7 days... high intensity exercise** |  |  |  |  | 0.723 |
| No | 14 (77.8%) | 24 (82.8%) | 21 (80.8%) | 1 (50.0%) |  |
| Yes | 4 (22.2%) | 5 (17.2%) | 5 (19.2%) | 1 (50.0%) |  |

Note: CHF, Chronic Heart Failure; BMI, Body Mass Index; CESD, Center for Epidemiologic Studies Depression Scale; ^A^ diabetes, heart attack, hypertension/high blood pressure, stroke, CHF; ^B^ by design of our sample there are no cancer in control group
