# Supplementary material for: Trajectories in muscular strength and physical function among men with and without prostate cancer in the health aging and body composition study
Source: PLoS One. 2020 Feb 13;15(2):e0228773. doi: 10.1371/journal.pone.0228773 (PMC7017990; doi:10.1371/journal.pone.0228773)
Supplement: S3 Table — (DOCX) [file pone.0228773.s003.docx]

### S3 Table. Change in quad strength at 3-4 year follow-up visit

|  | **Decreasing**  **Mean (SD) or n (%)** | **Consistently Low**  **Mean (SD) or n (%)** | **Consistently High**  **Mean (SD) or n (%)** | **p-value** |
| --- | --- | --- | --- | --- |
| **Age at index visit** | 77.1 (3.5) | 75.5 (3.0) | 74.3 (2.4) | 0.007 |
| **Years since index visit** |  |  |  | <.001 |
| **3** | 0 (0.0%) | 3 (18.8%) | 15 (68.2%) |  |
| **4** | 33 (100.0%) | 13 (81.3%) | 7 (31.8%) |  |
| **Race** |  |  |  | 0.570 |
| White | 15 (45.5%) | 9 (56.3%) | 13 (59.1%) |  |
| Black | 18 (54.5%) | 7 (43.8%) | 9 (40.9%) |  |
| **Education** |  |  |  | 0.549 |
| Less than HS | 5 (15.2%) | 3 (18.8%) | 4 (18.2%) |  |
| HS grad | 8 (24.2%) | 7 (43.8%) | 5 (22.7%) |  |
| Postsecondary | 20 (60.6%) | 6 (37.5%) | 13 (59.1%) |  |
| **Married** |  |  |  | 0.924 |
| No | 9 (27.3%) | 5 (31.3%) | 7 (31.8%) |  |
| Yes | 24 (72.7%) | 11 (68.8%) | 15 (68.2%) |  |
| **Diabetes** |  |  |  | 0.245 |
| No | 25 (75.8%) | 15 (93.8%) | 16 (72.7%) |  |
| Yes | 8 (24.2%) | 1 (6.3%) | 6 (27.3%) |  |
| **Heart Attack** |  |  |  | 0.289 |
| No | 31 (93.9%) | 15 (93.8%) | 18 (81.8%) |  |
| Yes | 2 (6.1%) | 1 (6.3%) | 4 (18.2%) |  |
| **Hypertension/High BP** |  |  |  | 0.666 |
| No | 14 (42.4%) | 8 (50.0%) | 12 (54.5%) |  |
| Yes | 19 (57.6%) | 8 (50.0%) | 10 (45.5%) |  |
| **Stroke** |  |  |  | 0.514 |
| No | 32 (97.0%) | 15 (93.8%) | 22 (100.0%) |  |
| Yes | 1 (3.0%) | 1 (6.3%) | 0 (0.0%) |  |
| **CHF** |  |  |  | 0.181 |
| No | 33 (100.0%) | 15 (93.8%) | 21 (100.0%) |  |
| Yes | 0 (0.0%) | 1 (6.3%) | 0 (0.0%) |  |
| **Number of Comorbidities** ^A^ | 0.9 (0.6) | 16: 0.8 (0.9) | 22: 0.9 (0.8) | 0.764 |
| **Arthritis** |  |  |  | 0.816 |
| No | 17 (51.5%) | 8 (50.0%) | 13 (59.1%) |  |
| Yes | 16 (48.5%) | 8 (50.0%) | 9 (40.9%) |  |
| **Cancer** ^B^ |  |  |  | 0.775 |
| No | 26 (78.8%) | 13 (81.3%) | 19 (86.4%) |  |
| Yes | 7 (21.2%) | 3 (18.8%) | 3 (13.6%) |  |
| **BMI** | 26.6 (4.0) | 26.4 (3.5) | 26.6 (3.0) | 0.988 |
| **% Body Fat** | 29.3 (3.5) | 29.7 (3.9) | 28.6 (3.9) | 0.654 |
| **Lean Body Mass (Kg)** | 53.8 (82.2) | 52.1 (67.4) | 54.9 (56.9) | 0.501 |
| **CESD** | 5.4 (4.8) | 12.3 (10.7) | 4.1 (4.7) | 0.056 |
| **Falls in last 12 months** |  |  |  | 0.321 |
| No | 23 (69.7%) | 13 (81.3%) | 19 (86.4%) |  |
| Yes | 10 (30.3%) | 3 (18.8%) | 3 (13.6%) |  |
| **Easy walking a quarter mile** |  |  |  | 0.824 |
| No | 3 (9.1%) | 1 (6.3%) | 1 (4.8%) |  |
| Yes | 30 (90.9%) | 15 (93.8%) | 20 (95.2%) |  |
| **Easy lifting/carrying 10 pounds** |  |  |  | 0.965 |
| No | 2 (6.1%) | 1 (6.3%) | 1 (4.5%) |  |
| Yes | 31 (93.9%) | 15 (93.8%) | 21 (95.5%) |  |
| **Past 12 months... high intensity exercise** |  |  |  | 0.590 |
| No | 22 (66.7%) | 12 (75.0%) | 13 (59.1%) |  |
| Yes | 11 (33.3%) | 4 (25.0%) | 9 (40.9%) |  |
| **Past 7 days... high intensity exercise** |  |  |  | 0.234 |
| No | 24 (72.7%) | 15 (93.8%) | 17 (77.3%) |  |
| Yes | 9 (27.3%) | 1 (6.3%) | 5 (22.7%) |  |

Note: CHF, Chronic Heart Failure; BMI, Body Mass Index; CESD, Center for Epidemiologic Studies Depression Scale; ^A^ diabetes, heart attack, hypertension/high blood pressure, stroke, CHF; ^B^ by design of our sample there are no cancer in control group
