# Supplementary material for: Trajectories in muscular strength and physical function among men with and without prostate cancer in the health aging and body composition study
Source: PLoS One. 2020 Feb 13;15(2):e0228773. doi: 10.1371/journal.pone.0228773 (PMC7017990; doi:10.1371/journal.pone.0228773)
Supplement: S4 Table — (DOCX) [file pone.0228773.s004.docx]

### S4 Table. Change in 20 m walking speed at 3-4 year follow-up visit

|  | **Decreasing**  **Mean (SD) or n (%)** | **Consistently Low**  **Mean (SD) or n (%)** | **Consistently High**  **Mean (SD) or n (%)** | **Increasing**  **Mean (SD) or n (%)** | **p-value** |
| --- | --- | --- | --- | --- | --- |
| **Age at index visit** | 75.8 (3.2) | 75.7 (3.0) | 76.2 (3.0) | 76.5 (4.8) | 0.934 |
| **Years since index visit** |  |  |  |  | 0.442 |
| **3** | 1 (2.0%) | 1 (10.0%) | 0 (0.0%) | 0 (0.0%) |  |
| **4** | 49 (98.0%) | 9 (90.0%) | 10 (100.0%) | 7 (100.0%) |  |
| **Race** |  |  |  |  | 0.351 |
| White | 25 (50.0%) | 3 (30.0%) | 6 (60.0%) | 5 (71.4%) |  |
| Black | 25 (50.0%) | 7 (70.0%) | 4 (40.0%) | 2 (28.6%) |  |
| **Education** |  |  |  |  | 0.544 |
| Less than HS | 11 (22.0%) | 1 (10.0%) | 1 (10.0%) | 1 (14.3%) |  |
| HS grad | 13 (26.0%) | 4 (40.0%) | 1 (10.0%) | 3 (42.9%) |  |
| Postsecondary | 26 (52.0%) | 5 (50.0%) | 8 (80.0%) | 3 (42.9%) |  |
| **Married** |  |  |  |  | 0.805 |
| No | 16 (32.0%) | 2 (20.0%) | 4 (40.0%) | 2 (28.6%) |  |
| Yes | 34 (68.0%) | 8 (80.0%) | 6 (60.0%) | 5 (71.4%) |  |
| **Diabetes** |  |  |  |  | 0.667 |
| No | 37 (74.0%) | 9 (90.0%) | 8 (80.0%) | 6 (85.7%) |  |
| Yes | 13 (26.0%) | 1 (10.0%) | 2 (20.0%) | 1 (14.3%) |  |
| **Heart Attack** |  |  |  |  | 0.956 |
| No | 46 (92.0%) | 9 (90.0%) | 9 (90.0%) | 6 (85.7%) |  |
| Yes | 4 (8.0%) | 1 (10.0%) | 1 (10.0%) | 1 (14.3%) |  |
| **Hypertension/High BP** |  |  |  |  | 0.604 |
| No | 24 (48.0%) | 6 (60.0%) | 4 (40.0%) | 2 (28.6%) |  |
| Yes | 26 (52.0%) | 4 (40.0%) | 6 (60.0%) | 5 (71.4%) |  |
| **Stroke** |  |  |  |  | 0.769 |
| No | 47 (95.9%) | 10 (100.0%) | 10 (100.0%) | 7 (100.0%) |  |
| Yes | 2 (4.1%) | 0 (0.0%) | 0 (0.0%) | 0 (0.0%) |  |
| **CHF** |  |  |  |  | 0.019 |
| No | 49 (100.0%) | 10 (100.0%) | 10 (100.0%) | 6 (85.7%) |  |
| Yes | 0 (0.0%) | 0 (0.0%) | 0 (0.0%) | 1 (14.3%) |  |
| **Number of Comorbidities** ^A^ | 50: 0.9 (0.7) | 10: 0.6 (0.7) | 10: 0.9 (0.6) | 7: 1.1 (1.1) | 0.491 |
| **Arthritis** |  |  |  |  | 0.017 |
| No | 26 (52.0%) | 1 (10.0%) | 8 (80.0%) | 4 (57.1%) |  |
| Yes | 24 (48.0%) | 9 (90.0%) | 2 (20.0%) | 3 (42.9%) |  |
| **Cancer** ^B^ |  |  |  |  | 0.139 |
| No | 42 (84.0%) | 10 (100.0%) | 8 (80.0%) | 4 (57.1%) |  |
| Yes | 8 (16.0%) | 0 (0.0%) | 2 (20.0%) | 3 (42.9%) |  |
| **BMI** | 26.6 (3.5) | 26.6 (4.2) | 26.4 (3.4) | 24.9 (3.2) | 0.675 |
| **% Body Fat** | 28.9 (4.1) | 28.8 (3.0) | 29.7 (3.1) | 28.9 (3.5) | 0.931 |
| **Lean Body Mass (Kg)** | 54.2 (71.1) | 54.3 (63.6) | 52.2 (74.7) | 50.8 (65.0) | 0.583 |
| **CESD** | 5.0 (5.2) | 4.0 (.) | 5.1 (5.2) | 8.7 (8.1) | 0.719 |
| **Falls in last 12 months** |  |  |  |  | 0.479 |
| No | 38 (76.0%) | 8 (80.0%) | 7 (70.0%) | 7 (100.0%) |  |
| Yes | 12 (24.0%) | 2 (20.0%) | 3 (30.0%) | 0 (0.0%) |  |
| **Easy walking a quarter mile** |  |  |  |  | 0.662 |
| No | 4 (8.2%) | 1 (10.0%) | 0 (0.0%) | 0 (0.0%) |  |
| Yes | 45 (91.8%) | 9 (90.0%) | 10 (100.0%) | 7 (100.0%) |  |
| **Easy lifting/carrying 10 pounds** |  |  |  |  | 0.640 |
| No | 3 (6.0%) | 0 (0.0%) | 0 (0.0%) | 0 (0.0%) |  |
| Yes | 47 (94.0%) | 10 (100.0%) | 10 (100.0%) | 7 (100.0%) |  |
| **Past 12 months... high intensity exercise** |  |  |  |  | 0.060 |
| No | 35 (70.0%) | 9 (90.0%) | 7 (70.0%) | 2 (28.6%) |  |
| Yes | 15 (30.0%) | 1 (10.0%) | 3 (30.0%) | 5 (71.4%) |  |
| **Past 7 days... high intensity exercise** |  |  |  |  | 0.806 |
| No | 41 (82.0%) | 9 (90.0%) | 8 (80.0%) | 5 (71.4%) |  |
| Yes | 9 (18.0%) | 1 (10.0%) | 2 (20.0%) | 2 (28.6%) |  |
| **Follow-up time (years from index to follow-up)** | 4.0 (0.2) | 3.8 (0.3) | 4.0 (0.1) | 4.0 (0.1) | 0.014 |

Note: CHF, Chronic Heart Failure; BMI, Body Mass Index; CESD, Center for Epidemiologic Studies Depression Scale; ^A^ diabetes, heart attack, hypertension/high blood pressure, stroke, CHF; ^B^ by design of our sample there are no cancer in control group
